# Genome-wide genetic structure and selection signatures for color in 10 traditional Chinese yellow-feathered chicken breeds

**DOI:** 10.1186/s12864-020-6736-4

**Published:** 2020-04-20

**Authors:** Xunhe Huang, Newton O. Otecko, Minsheng Peng, Zhuoxian Weng, Weina Li, Jiebo Chen, Ming Zhong, Fusheng Zhong, Sihua Jin, Zhaoyu Geng, Wei Luo, Danlin He, Cheng Ma, Jianlin Han, Sheila C. Ommeh, Yaping Zhang, Xiquan Zhang, Bingwang Du

**Affiliations:** 1 Guangdong Provincial Key Laboratory of Conservation and Precision Utilization of Characteristic Agricultural Resources in Mountainous Areas, Guangdong Innovation Centre for Science and Technology of Wuhua Yellow Chicken, School of Life Science of Jiaying University, Meizhou, 514015 China; 2 State Key Laboratory of Genetic Resources and Evolution and Yunnan Laboratory of Molecular Biology of Domestic Animals, Kunming Institute of Zoology, Chinese Academy of Sciences, Kunming, 650223 China; 3 Kunming College of Life Science, University of Chinese Academy of Sciences, Kunming, 650204 China; 4 College of Animal Science and Technology, Hunan Agricultural University, Changsha, 410128 China; 5 College of Animal Science and Technology, Anhui Agricultural University, Hefei, 230036 China; 6 College of Animal Sciences, South China Agricultural University, Guangzhou, 510642 China; 7 CAAS-ILRI Joint Laboratory on Livestock and Forage Genetic Resources, Institute of Animal Science, Chinese Academy of Agricultural Sciences (CAAS), Beijing, 100193 China; 8 International Livestock Research Institute (ILRI), Nairobi, 30709-00100 Kenya; 9 Animal Biotechnology Group, Institute For Biotechnology Research, Jomo Kenyatta University of Agriculture and Technology, Nairobi, 62000-00200 Kenya; 10 State Key Laboratory for Conservation and Utilization of Bio-resources in Yunnan, Yunnan University, Kunming, 650091 China; 11 Center for Excellence in Animal Evolution and Genetics, Chinese Academy of Sciences, Kunming, 650223 China

**Keywords:** Yellow, Chicken, Genome, *BCDO2*, Breeding, Color, Genetic diversity

## Abstract

**Background:**

Yellow-feathered chickens (YFCs) have a long history in China. They are well-known for the nutritional and commercial importance attributable to their yellow color phenotype. Currently, there is a huge paucity in knowledge of the genetic determinants responsible for phenotypic and biochemical properties of these iconic chickens. This study aimed to uncover the genetic structure and the molecular underpinnings of the YFCs trademark coloration.

**Results:**

The whole-genomes of 100 YFCs from 10 major traditional breeds and 10 Huaibei partridge chickens from China were re-sequenced. Comparative population genomics based on autosomal single nucleotide polymorphisms (SNPs) revealed three geographically based clusters among the YFCs. Compared to other Chinese indigenous chicken genomes incorporated from previous studies, a closer genetic proximity within YFC breeds than between YFC breeds and other chicken populations is evident. Through genome-wide scans for selective sweeps, we identified RALY heterogeneous nuclear ribonucleoprotein (*RALY*), leucine rich repeat containing G protein-coupled receptor 4 (*LGR4*), solute carrier family 23 member 2 (*SLC23A2*), and solute carrier family 2 member 14 (*SLC2A14*), besides the classical beta-carotene dioxygenase 2 (*BCDO2*), as major candidates pigment determining genes in the YFCs.

**Conclusion:**

We provide the first comprehensive genomic data of the YFCs. Our analyses show phylogeographical patterns among the YFCs and potential candidate genes giving rise to the yellow color trait of the YFCs. This study lays the foundation for further research on the genome-phenotype cross-talks that define important poultry traits and for formulating genetic breeding and conservation strategies for the YFCs.

## Background

Different cultures and ethnicities around the globe have developed unique cuisines, into which chickens are incorporated in diverse ways. Chicken consumption is popular globally, with the preference for chicken meat eclipsing that of red meat [1, 2]. Yellow-feathered chickens, otherwise known as “three-yellow chickens” because of their characteristic yellow beak, feathers, and feet [1], and herein abbreviated as YFCs, are a favorite choice for traditional broths and soups in Asian countries, particularly in Korea and southern China. YFCs have been described in the ancient Chinese agricultural text “*Qimin Yaoshu*” written around 540 C.E [3], and their importance is evidenced by the incredible leap in their demand. For instance, the production of YFC meat in China reached 4445 kt in 2015, representing 31.8% of the national broiler meat yields [4]. The YFCs’ unique meat flavor and color appeal are important factors driving this strong consumer preference. In addition to serving as a traditional nutritional and commercial mainstay for millions of people living in China and its purlieus, YFCs are reported to have contributed to the recent breeding of European chickens [5], indicating a broadening utility of the YFCs. At the present, more than 15 traditional breeds of YFCs are dispersed widely in China [6]. However, these attributes are threatened by the aggressive genetic selection for rapid growth and high feed conversion efficiency in China and other Asian countries [7]. Previous research on YFCs primarily focused on understanding the chemical properties of meat and soups [2, 8–10], or their genetic diversity compared to commercial breeds using low-density markers [1, 11, 12]. Hardly any genome-wide investigations of the population structure and genetic basis of the unique YFC phenotypic traits have been conducted, a major drawback in rational improvement and conservation of these chickens.

In this study, we sought to accomplish an extensive sequencing of YFC populations across China to support their in-depth studies into their evolutionary biology. We also aimed to implement comparative population genetic analyses to determine the genetic structure of the YFCs and retrieve the footprints of selection for their unique color property. This study provides vital resources and insights to facilitate effective avicultural strategies.

## Results

### Characteristics of the genome datasets

**Fig. 1:**
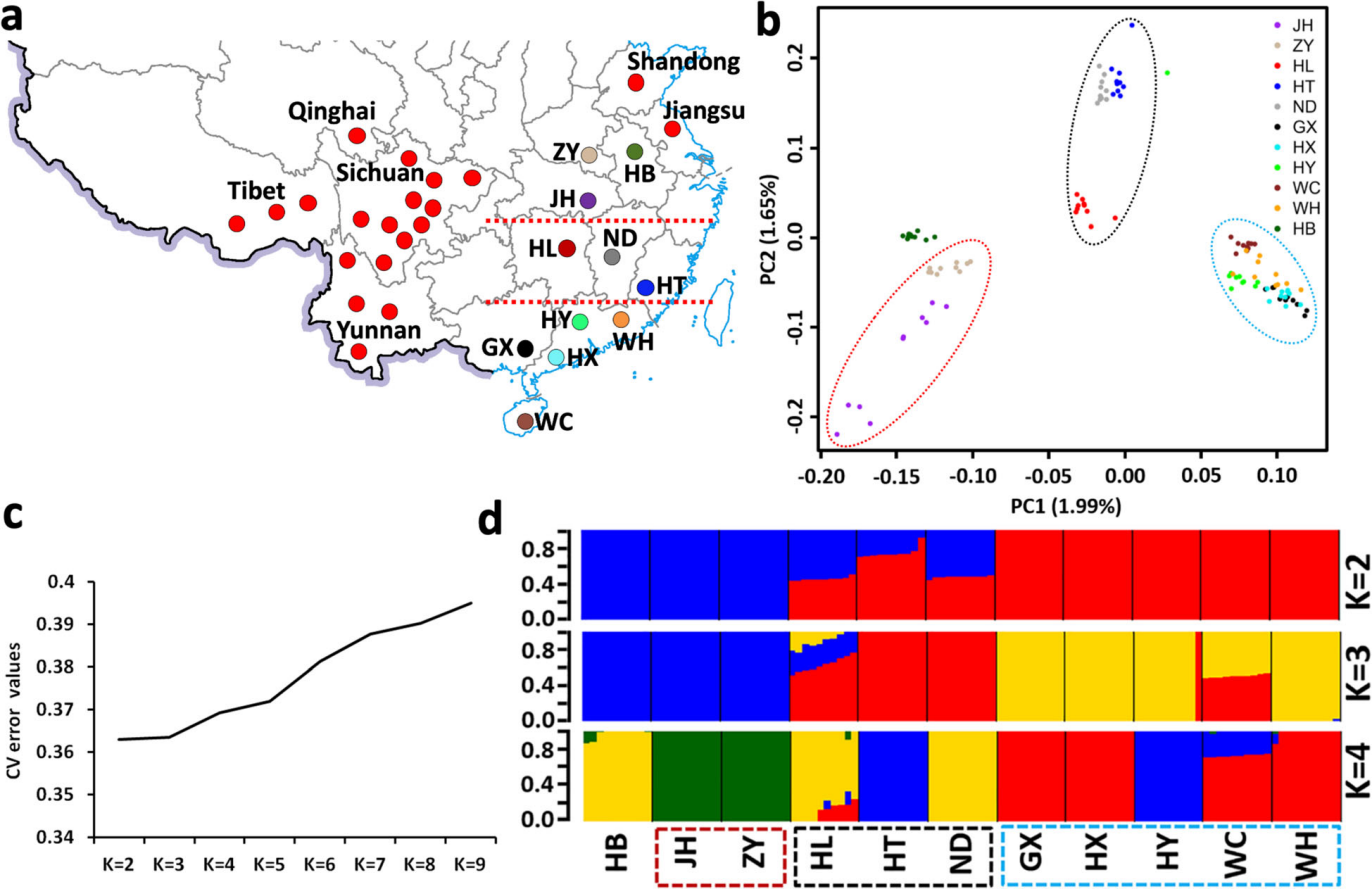
Population genomic analysis of the YFCs. **a** Sampling map (adapted from http://bzdt.ch.mnr.gov.cn/) showing the geographical locations of all chicken breeds/populations. The newly described chicken breeds are noted by their respective population’s IDs, while red cycles indicate chicken populations retrieved from previous studies [13, 14]. The two populations from Shandong and Jiangsu provinces are Yuanbao bantams. The dotted horizontal lines demarcate the three population clusters. **b** Principal component analysis (PCA) of all 110 chickens sequenced in this study. YFCs’ clustering patterns are highlighted by dotted red (northern cluster), black (central cluster), and blue (southern cluster) circles. Breed code: HB, Huaibei partridge; ZY, Zhengyang Yellow; JH, Jianghan; ND, Ningdu Yellow; HL, Huanglang; HT, Hetian; WH, Wuhua Yellow; HY, Huiyang bearded; HX, Huaixiang; GX, Guangxi Yellow; WC, Wenchang. **c-d** ADMIXTURE analysis for *K* = 2, *K* = 3, and *K* = 4

We performed an initial in-depth characterization of the genomes of the 100 YFCs from 10 different breeds and 10 Huaibei (HB) partridge chickens (used for comparisons) sequenced in this study **(**Fig. 1a; Additional file 1**)**. An average of 86,155,900 clean reads per genome are obtained after quality control protocols, which were then aligned to the reference genome, yielding a mean mapping rate at 87.12% (Additional files 2 and 3, Additional file 4: Fig. S1). The total average base coverage across the genome is 96.35% at a sequencing depth target of 1X, 86.81% at 4X, 41.87% at 10X, and down to 0.36% at 30X (Additional file 4: Fig. S2). The average number of nucleotides in each genome is 11,916,810,290 after filtration, with an average GC content at 44.53% (Additional file 5).

For comparative analyses, we merged the 100 YFC and 10 Huaibei partridge chicken genomes with 104, 10, and 1 previously published Chinese chicken, red junglefowl (*Gallus gallus*; RJF), and green junglefowl (*Gallus varius*; GVF) genomes, respectively, retaining a total of 3,065,814 common autosomal single nucleotide polymorphisms (SNPs).

### Genome variants in the yellow-feathered chickens

After filtration, 16,817,111 single nucleotide polymorphisms (SNPs) and 1,289,024 InDels (insertion or deletion of bases) (≤ 50 bp) were retained. The structural variations (SVs) and the increase or decrease of the copy number of large (> 1 kb) genomic fragments were analyzed. All these genomic variants in the newly generated dataset are summarized in Additional file 4: Fig. S3. Briefly, most of the SNPs are located in intergenic followed by intronic genomic regions (Additional file 4: Fig. S4a). Those located within coding sequences are mainly associated with synonymous or nonsynonymous coding attributes (Additional file 4: Fig. S4b). There are more transitions (11,943,736; 71.02%) than transversions (4,873,375; 28.98%) in the dataset. G- > A and C- > T substitutions are the common transitions at 28% while A- > G and T- > C substitutions are around 21% (Additional file 4: Fig. S5). Different transversions show a low but relatively uniform distribution rate in the dataset. The total average ratio of transitions to transversions is 2.53 (Additional file 6). Analysis of the heterogeneity of clean SNPs shows that about 2,033,275 and 2,259,628 SNPs per genome are homogenous and heterozygous hybrids, respectively (Additional file 7).

Among the high quality InDels, there are more deletions than insertions (785,806 (60.96%) versus 503,218 (39.04%)). The genomic locations of these InDels are summarized in Additional file 4: Fig. S6a, where most InDels are located in non-coding (i.e. intergenic and intronic) regions. Additionally, the four most common genomic consequences of the InDels include frameshift or non-frameshift insertions and deletions (Additional file 4: Fig. S6b).

Besides SNPs and InDels, SVs, which represent a large range of chromosomal variations encompassing large genomic regions have been characterized. These include large fragment deletions (DEL), insertions (INS), inversions (INV), translocations, and duplications [15, 16]. Intrachromosomal translocations (65%) and deletions (26%) are predominant in the dataset, while inversions and interchromosomal translocations are present in lower proportions (Additional file 4: Fig. S7a, Additional file 8). Analysis of copy number variations (CNVs; 95,918 in total), divided into deletions and duplications, reveals an overall higher proportion of deletions (56.5%) than duplications (43.5%) (Additional file 4: Fig. S7b, Additional file 9).

### Population structure

Principal component Analysis (PCA) was performed for all the 100 YFC genomes, revealing a general separation of YFCs from Henan (Zhengyang, ZY) and Hubei (Jianghan, JH) (Fig. 1b) into a northern cluster. The YFCs from Guangxi (Guangxi Yellow, GX), Guangdong (Huaixiang, HX, Huiyang bearded, HY, and Wuhua Yellow, WH), and Hainan (Wenchang, WC) form a southern cluster, while those from Hunan (Huanglang, HL), Jiangxi (Ningdu Yellow, ND), and Fujian (Hetian, HT) group into a central cluster. This finding is supported by ADMIXTURE analysis (Fig. 1c and d). At the lowest cross validation error value, corresponding to *K* = 2, the northern (blue) and southern (red) clusters show a complete separation, whereas the central cluster exhibits a signal of admixture with the northern and southern clusters. These three clusters were verified when *K* = 3, with HL and WC showing admixed ancestries. When *K* = 4, both HT and HY harbor the same ancestry component, which also contribute to WC.

**Fig. 2:**
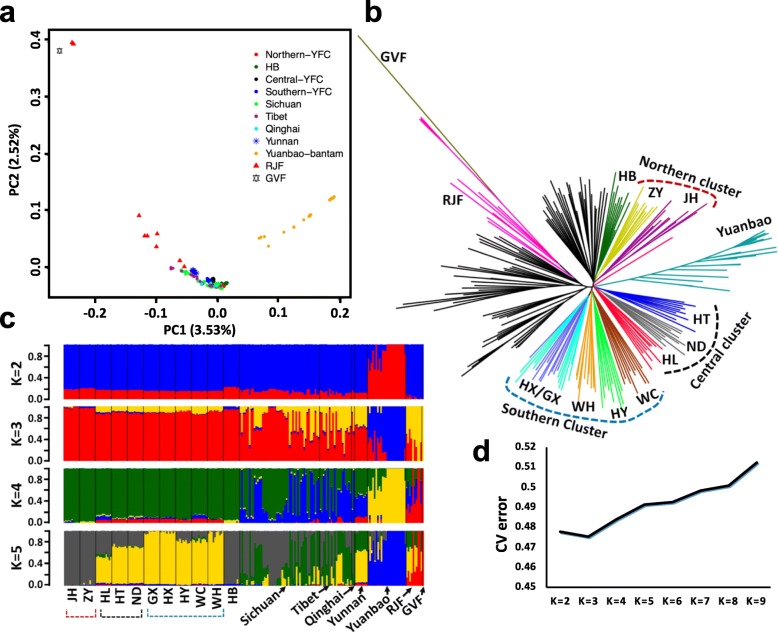
Population genomic analysis of YFCs in the context of other Chinese indigenous chickens. **a** PCA showing the evolutionary relationships among YFCs, other Chinese indigenous chickens, red junglefowl (RJF), and green junglefowl (GVF). **b** Neighbor joining tree including all chickens and RJF (root). The tree was viewed and edited using FigTree software (v1.4.3). **c**-**d** ADMIXTURE analysis for *K* = 2 up to *K* = 5. The lowest cross validation error is observed when *K* = 2

We implemented comparative population genetic analyses the YFCs against other indigenous chickens from China, RJF, and GVF. In the PCA (Fig. 2a), YFCs tend to cluster together and appear to be in close proximity to HB partridge chickens and a few indigenous chickens from Sichuan and Tibet. These patterns imply a close congruity in the total genomic architecture of the YFCs. Yuanbao bantams form a distant cluster from the YFCs and other chickens, underscoring the genomic effects of differential breeding trajectories [13, 17]. Neighbor joining (NJ) phylogeny (Fig. 2b) and ADMIXTURE (Fig. 2c and d) corroborates the findings of the PCA and further clarifies the northern, central, and southern YFC clustering pattern inferred from Fig. 1b-d.

### Detection of selective sweeps

**Fig. 3:**
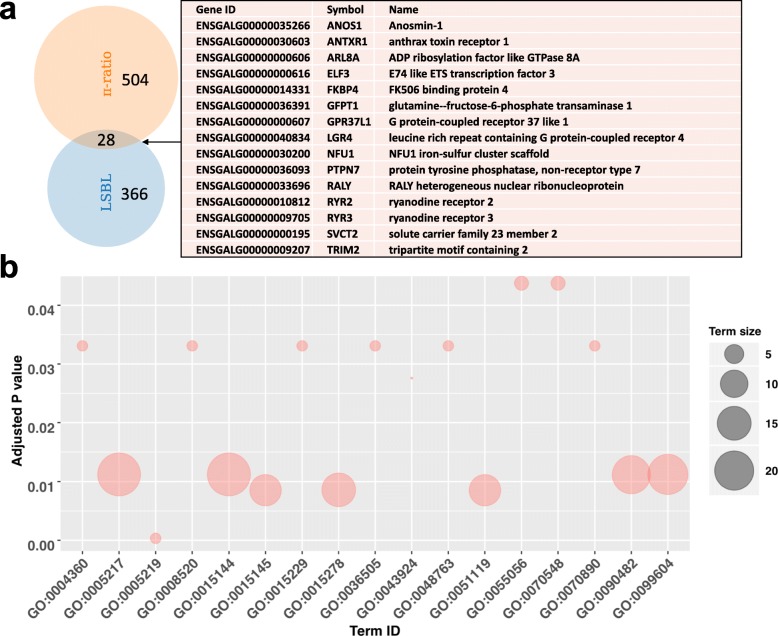
Candidate positively selected genes (PSGs). **a** PSGs in the top 1% windows of LSBL and π-ratio genome selection tests. The identities of annotated genes overlapping in both tests are shown. **b** Summary of the functional enrichment analysis of the 28 overlapping PSGs. Only terms with adjusted *P* values less than 0.05 are shown

**Fig. 4:**
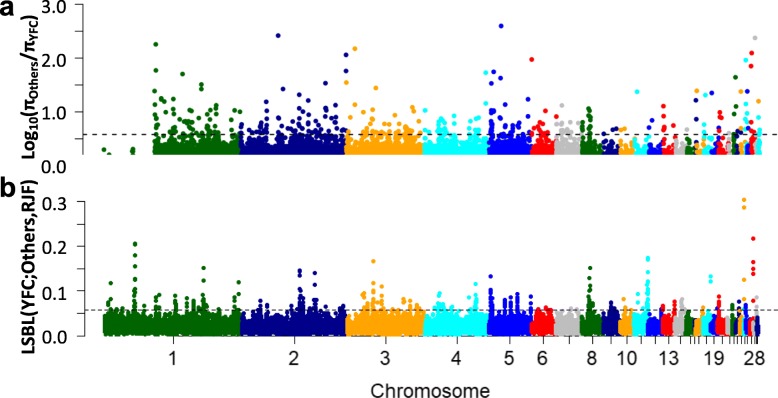
Genome-wide distributions of selection signals. **a** π-ratio contrasting Yellow-feathered chickens (YFC) against chicken with non-yellow (black) phenotypes, denoted as Others. **b** Locus-specific branch length (LSBL) analysis contrasting YFC against non-yellow chicken with red junglefowl (RJF) outgroup. The horizontal dotted lines represent the top 1% cut-off

Genome-wide scans for signals of selection attributable to the YFCs phenotype identified 268 analytical windows within the top 1% of the Locus-specific branch length (LSBL) test, and 370 windows in the π-ratio test (Additional files 10 and 11). These correspond to 366 and 504 positively selected genes (PSGs), respectively. A total of 28 PSGs were concurrently identified in the top 1% by the two selection tests (Fig. 3a). This is a relatively small overlap, possibly owing to the differences in the selection tests. Among the 28 genes are genes that are associated with pigmentation including: RALY heterogeneous nuclear ribonucleoprotein (*RALY*), leucine rich repeat containing G protein-coupled receptor 4 (*LGR4*), ryanodine receptor 2 (*RYR2*), *RYR3*, solute carrier family 23 member 2 (*SLC23A2*), and *SLC2A14*. Functional enrichment assessment showed significant gene ontology (GO) terms including vitamin transport activity (GO:0090482; Fig. 3b), intersecting with *SLC23A2* and *SLC2A14*, which play roles in pigmentation. There are additional genes above the top 1% significance threshold in either of the selection tests. These genes are important for understanding the color trait and other properties of interest like meat quality of the YFCs. They include *BCDO2*, *IL-18*, *FBXO5*, *COL1A2*, *COL4A2*, *COL6A1*, *COL6A2* in LSBL; and *GDF8*, *HSPA5*, *SHISA9*, *COL4A1*, and *COL23A1* in the π-ratio test (Fig. 4).

### *BCDO2* haplotype differentiation

**Fig. 5:**
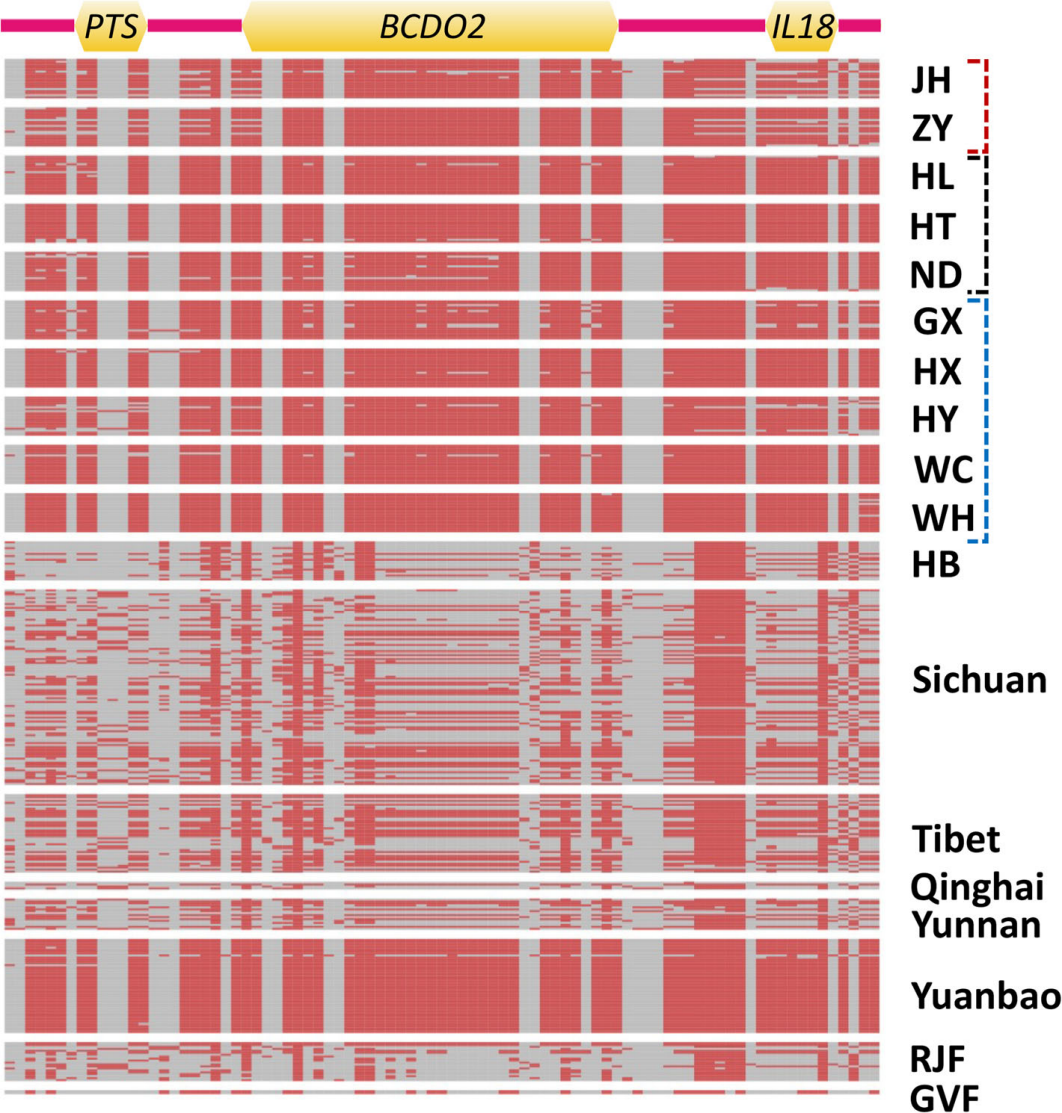
Genomic diversity and *BCDO2* gene structure of YFCs and other chickens. Haplotype pattern analysis of the yellow skin gene, *BCDO2* across different populations separated by white gaps. Immediate flanking genes are indicated. Grey color denotes reference alleles while Indian red indicates alternative alleles. Initials represent chicken breeds/population names as defined in the methods. The three YFC clusters are indicated by red, black, and blue boxes representing northern, central, and southern clusters, respectively

*BCDO2* gene is a classical yellow color gene in chicken. We investigated its haplotype structure, also encompassing the proximal flanking genes. *BCDO2* showed a homogenous haplotype pattern across the 10 YFC breeds (Fig. 5). Interestingly, Yuanbao breed also bears the same pattern as the YFCs. On the other hand, HB partridge chickens, which initially showed a close genomic proximity to the northern cluster YFCs (Fig. 1), clearly exhibits a synonymous *BCDO2* haplotype pattern to the other Chinese indigenous chickens rather than to the YFCs (Fig. 5). Overall, the haplotype differentiation pattern of *BCDO2* and its flanking genes (*IL18* and *PTS*) is consistent with the selection of these genes as candidate PSGs for the yellow pigmentation phenotype.

## Discussion

We provide the first comprehensive whole-genome sequencing data and genomic variants for the YFCs. We also describe the genetic structure and molecular background of the distinguished color phenotype of these chickens. YFCs are a traditional nutritional and commercial mainstay for millions of people living in China and its purlieus, and are believed to have contributed to the recent breeding of European chickens [5]. Next generation sequencing has augmented scientific research into the molecular foundations of various complex phenotypic poultry traits such as body size in chicken [13], body size and plumage color in ducks [18], as well as maturation and plumage color in domestic quails [19], among others. In this study, we characterize not only the SNPs in the genomes of the YFCs but also other variants including InDels, structural variations (SV), and copy number variations (CNV) to facilitate research of these chickens. Particularly, SVs are increasingly gaining research interest as they can lead to the birth of new genes, change gene copy number as well as their expression profiles, eventually affecting phenotypic evolution and adaptation of organisms to local environments [20–23], hence will be an important resource to extend the SNP-based genetic analyses [24, 25]. Similarly, CNVs are linked with phenotypic evolution and have supported high-impact evolutionary investigations on complex diseases and economically important traits [26, 27]. For instance, in chicken, sequence duplication near the first intron of *SOX5* gene is linked with the chicken pea-comb trait [28], an inverted duplication covering *EDN3* gene leads to dermal hyperpigmentation [29], and a partial duplication of *PRLR* gene is associated with late feathering [30].

Our current comparative population genomics analysis was anchored on genome-wide SNPs of the YFCs, other indigenous chickens, and wild ancestors. Population structure analysis revealed an overall distinctive genomic architecture of the YFCs from other Chinese indigenous chickens (PCA and NJ phylogenetic tree). Interestingly, a three-way sub-clustering pattern is consistent in PCA, ADMIXTURE, and NJ phylogenetic tree and amazingly mirrors the geographical distributions of the YFCs. The 10 YFC populations divide into northern, central and southern clusters, agreeing with the trends earlier proposed by microsatellite-based studies of chickens from these regions of China [1, 11, 12]. This sub-structuring may be reflective of some extent of differential exchange of genetic materials in neighboring locations, breeding histories, or natural and artificial selection drivers as described in several chicken populations [17, 27]. This explains the existence of genomic grouping among populations with close phenotypic appearances such as the YFCs. A crucial point to note is the signals of admixture at *K* = 3 and 4 in the ADMIXTURE analysis. Hetian (HT) and Huiyang bearded (HY) YFCs are historically ascribed to the Hakka Chinese [6] who are thought to have immigrated from northern China, and have preserved their distinguished cultures, languages [31], and even genetic attributes [32]. Wenchang (WC) chickens are reported to have originated from crossbreeding of chickens brought into Hainan Province by people (including the Hakka) from Guangdong and Fujian Provinces [6]. The results of PCA and ADMIXTURE (*K* = 2 and 3) suggest that the Huaibei (HB) partridge chickens have a close relationship with YFCs of the northern cluster, consistent with their geographical proximity. Nevertheless, it is incomprehensible that HB, Huanglang (HL), and Ningdu Yellow (ND) shared dominant ancestry component at *K* = 4. Compared to other indigenous Chinese chickens, the YFCs tend have a closer genetic semblance among themselves than with other chickens, inferring a possible overriding effect of selection for the outstanding phenotypic traits of the YFCs.

Fundamental to the genomic selection scans in this study is the identification of *RALY*, *LGR4*, *RYR2*, *RYR3*, and *SLC23A2* as well as its related homologue, *SLC2A14*. These genes stood out as candidate genes under selection in the YFCs, having significant signals both in LSBL and π-ratio scans. There is a known epistatic relationship bringing together *RALY*, *ASIP*, and *MC1R* [33]. *ASIP* gene codes for agouti-signaling protein, which antagonizes the α-MSH hormone (melanocyte stimulating hormone) for the melanocyte-1 receptor *(MC1R*) counteracting the production of eumelanin (black/brown melanin) and favoring the synthesis of pheomelanin (yellow/red melanin) [34]. Both *ASIP* and *MC1R* are genes which continue to be synonymous with nearly all studies on pigmentation in mammalian and avian species [35–39]. Interestingly, it has been demonstrated that a > 90 kb deletion upstream of avian *ASIP*, encompassing portions of the *RALY* locus, places *ASIP* under the regulatory control of *RALY* promoter [40]. The resulting up-regulation of *ASIP* underlies the yellow feather phenotype in quails and is interestingly associated with down-regulation of *SLC24A5* [40]. *SLC24A5* is an important gene in pigmentation whose roles in eumelanogenesis has been clearly demonstrated in both human and zebrafish [41]. We detected two members of the solute carrier family (SLC), *SLC23A2* and *SLC2A14*. *SLC23A2* is a major mediator of the transport of ascorbic acid, an indispensable metabolite that is fundamental for survival [42]. Anomalies in the availability of this vitamin have been associated with neonatal jaundice and yellow chromophore in eye lenses of human and humanized mouse model [43, 44]. It is key to note that although the selection of neither *ASIP* nor *MC1R* did not reach significance in our analyses, genes with which they are closely related, particularly *RALY* and SLC family homologues such as *SLC23A2* and *SLC2A14* point to the possibility of a gene network encompassing the PSGs identified in this study, working in conjunction with *ASIP* and *MC1R* in the determination of the yellow color trait of the YFCs.

From the selection sweep analysis, it was not a surprise to detect a strong selection for the *BCDO2* gene and a common genetic architecture of the gene among all the YFCs. Even the HB chickens which are phylogenetically and geographically close to the YFCs in the northern cluster were clearly distinguishable based on the *BCDO2* haplotype structure, depicting a possible marked differentiation of indigenous chickens at trait-linked genome compartment under selection pressure, despite likely closeness at the total genome level. Besides the YFCs, the *BCDO2* haplotype for yellow skin is also observed in Yuanbao chicken, which also have yellow skin, and at a low frequency in some indigenous chicken, consistent with the reporting of related haplotypes of this gene in southern China chickens [45].

Moreover, our results show some clues for meat quality which is a major economic feature in chicken production. *RYR2* and *RYR3* [46–48];* IL-18* [49];* FBXO5* [50]; *COL1A2*, *COL4A2*, *COL6A1*; *COL6A2*; *COL4A1*, and *COL23A1* [51]; *GDF8* [52]; *HSPA5* [49]; *SHISA9* [53]; all bearing strong selection signals in the YFCs, are important determinants of meat quality in domestic animals. These genes provide a foundation for understanding the meat properties of the YFCs, which would attract more concerns to investigate the detailed function roles in future studies.

## Conclusions

In summary, this study provides an invaluable resource for further research on the molecular mechanisms conferring complex traits that are of high economic and nutritional value. Through genomic insights regarding key genes behind the unique traits of YFCs and a comprehensive data resource, this study paves way for reconstructing the breeding history and formulating future conservation and breed improvement strategies for YFCs.

## Methods

### Samplings and sequencing of the yellow-feathered chickens

Unrelated chickens were identified with the help of pedigree records. Wing-vein blood samples were then collected by trained local veterinary personnel, from 100 birds of 10 YFC breeds, 10 chickens per breed. These breeds include Guangxi Yellow (GX), Hetian (HT), Huaixiang (HX), Huanglang (HL), Huiyang bearded (HY), Jianghan (JH), Ningdu Yellow (ND), Wenchang (WC), Wuhua Yellow (WH), and Zhengyang Yellow (ZY) (Additional file 1). Ten Huaibei partridge (HB) chickens were also sampled for comparison. Animal handling and experimentation was conducted according to the guidelines approved by the Animal Ethics Committee of Jiaying University and Kunming Institute of Zoology, Chinese Academy of Sciences. Genomic DNA was extracted using the standard phenol-chloroform method and checked for quantity and quality using agarose gel electrophoresis and NanoDrop spectrophotometer 2000 (NanoDrop, Wilmington, DE, USA). Whole genome sequencing for these 110 samples was carried out to a median depth of 12.22X (ranging from 9.34 to 18.66) (Additional file 1) using the Illumina HiSeq X10 platform at the Genedenovo Biotechnology Co., Ltd. (Guangzhou, China).

### Identification and annotation of whole-genome variants

To obtain high quality clean reads, stringent quality filtering procedures were applied as follows: 1) removing reads with ≥ 10% unidentified nucleotides (N); 2) removing reads with > 50% bases having phred scores of ≤ 20; and 3) removing reads aligned to the barcode adapter. The clean reads were then aligned to the chicken reference genome (*Galgal5*) [54] using the BWA-MEM alignment algorithm [55] implemented in the BWA [56] with options ‘mem 4 -k 32 -M’. Further quality control processes were performed using the SortSam and MarkDuplicates tools in the Picards package (picard-tools-1.56) to sort and remove possible duplicates in the aligned BAM files, and the RealignerTargetCreator, IndelRealigner, and BaseRecalibrator tools in the Genome Analysis Toolkit (GATK 2.6–4) [57] for local realignment and base quality recalibration. The bedtools software (v.2.25.0) [58] was employed to generate sequencing coverage statistics.

Variant calling was performed using the GATK’s Unified Genotyper. SNPs and InDels were filtered by the GATK’s VariantFiltration with options “-Window 4, -filter “QD < 2.0 || FS > 60.0 || MQ < 40.0 “, -G_filter “GQ < 20″”, excluding those exhibiting segregation distortions or sequencing errors. The ANNOVAR [59] was used to assign putative genomic positions of SNPs and InDels against the chicken gene database in ENSEMBL (release 92.5). The structural variations (SVs) in these 110 chicken genomes were assessed using the BreakDancer package (Max1.1.2.) [60, 61], and the CNVnator program (v.0.3.2) [62] was employed to classify copy number variations (CNVs).

### Analysis of population genetic structure

The evolutionary interactions among the 100 YFCs were examined using principal component analysis (PCA) following the GCTA approach [63] and maximum-likelihood-based ADMIXTURE [64] at *K* = 2 to 9. The PLINK package (v.1.90) was used to obtain pruned data with parameters “--indep-pairwise 50 10 0.1” [65] for the PCA and ADMIXTURE analyses. To perform a comparative analysis of the YFC genomes generated in this study against those of other chicken populations, 104 previously published whole genomes [13, 14] of Chinese indigenous chickens (Sichuan, *n* = 50; Tibet, *n* = 20; Qinghai, *n* = 2; Yunnan, *n* = 8; and Yuanbao bantams, *n* = 24), as well as 10 RJF and 1 GVF genomes were included. After merging our dataset of the 110 chicken genomes with the additional 115 genomes, 3,065,814 common SNPs were retained for subsequent analyses. The PCA was performed as stated above and a neighbor joining (NJ) tree rooted to RJF was constructed using the RapidNJ program [66] with 100 bootstrap replications.

### Genomic targets of selection in yellow-feathered chickens

To retrieve the genetic foundation for the outstanding phenotypic properties of the YFCs, we performed genome-wide scans for signals of selection using locus-specific branch length (LSBL) statistics [67] and π-ratio. Use of multiple statistical approaches helps to manage inherent differences of individual tests and increase the reliability of the selective sweep detection [68]. The comparative genomic analysis approach involved genomes of the YFCs against 24 chickens with contrasting non-yellow phenotypes (non-YFCs), i.e. black-phenotype Chinese chickens (five Emei black fowl, four Miyi fowl, five Muchuan black-bone fowl, and five Tuanfu black-bone fowl; Additional file 1) and RJF. In the LSBL, we computed LSBL(A;B,C) = (*F*_ST(AB)_ + *F*_ST(AC)_–*F*_ST(BC)_)/2 to assess the population differentiation between YFCs and other chickens, set as ‘YFCs;non-YFCs,RJF’. *F*_ST_ values were calculated as described elsewhere [69] with a 50-kb sliding window and 25-kb stepwise increments. π-ratio was performed by first calculating the genetic diversity (π) for YFCs and the 24 non-YFCs populations using VCFtools [70] in 50-kb windows with 25-kb stepwise increments, then computing π-ratio (π_non-YFCs_/π_YFCs_). An empirical cutoff of 99th percentile was used to retrieve candidate selective sweeps, which were then annotated using variant effect predictor (VEP) to identify the putative positively selected genes (PSGs) [71].

We performed functional enrichment analysis using g:Profiler [72] to obtain a global overview on the biological functions of the candidate PSGs with concordantly significant selection signals in the two genomic selection scans employed. A Benjamini-Hochberg false discovery rate (FDR) significance threshold was set at 0.05.

### Assessment of the classical yellow skin gene, *BCDO2*

*BCDO2* gene, located in chromosome 24: 6,110,301-6,130,965 reverse strand (*Galgal5*, [54]), is believed to be substantially associated with yellow skin pigmentation in chickens following a possible introgression from grey junglefowl (*Gallus sonneratii*) in South Asia [73]. We evaluated the haplotype variability of *BCDO2* gene and its flanking genes, a stretch of the genome from chromosome 24:6,105,000 - 6,145,000. Haplotypes for were phased using BEAGLE software (v.3.3.2) with the default parameters [74] and viewed as heatmaps. Gene structure representation was done using the Gene Structure Display Server (GSDS v.2) [75].

## Supplementary information

**Additional file 1.** Individual sample characteristics of all chickens sequenced in this study.

**Additional file 2.** Reads filtering statistics for the chicken sequenced in this study.

**Additional file 3.** Genome alignment statistics for all chickens sequenced in this study.

**Additional file 4: Fig. S1** Constitution of clean sequencing reads of all 110 chicken genomes produced in this study. **Fig. S2** Summary of the average sequencing coverage of all 110 chicken genomes generated in this study. **Fig. S3** Circos plot depicting the genomic variants landscape in each chromosome. **Fig. S4** Annotation of the clean genomic SNPs of all 110 chickens sequenced in this study. **Fig. S5** Transition-transversion analysis of the clean SNPs of all 110 chicken genomes sequenced in this study. **Fig. S6** Annotation of the clean InDels of all 110 chicken genomes sequenced in this study. **Fig. S7** Summary of the structural variations (SVs) and copy number variations (CNVs) in all 110 chicken genomes generated in this study.

**Additional file 5.** Base information statistics before and after quality filtering of all chicken genomes sequenced in this study.

**Additional file 6.** Individual transitional and transversional SNP statistics of all chickens sequenced in this study.

**Additional file 7.** Annotation of the hybrid status of SNPs in each chicken genome sequenced in this study.

**Additional file 8.** Annotation of structural variations in the chicken genomes sequenced in this study.

**Additional file 9.** Annotation of copy number variations in the chicken genomes sequenced in this study.

**Additional file 10.** The top 1% genomic windows in the selection scan by LSBL test.

**Additional file 11.** The top 1% genomic windows in the selection scan by π-ratio test.

## Data Availability

All new sequencing data generated in this study have been deposited in the NCBI sequence read archive (SRA) under accession number SRP155577. Further details are provided in Additional file 1. Additional requests can be channeled to the corresponding authors.

## Abbreviations

*BCDO2*: Beta-carotene dioxygenase 2
CNV: Copy number variation
FDR: False discovery rate
GO: Gene ontology
GVF: Green junglefowl
InDel: Insertion or deletion of bases
INS: Insertions
INV: Inversions
*LGR4*: Leucine rich repeat containing G protein-coupled receptor 4
LSBL: Locus-specific branch length
NJ: Neighbor joining
PCA: Principal component analysis
PSGs: Positively selected genes
*RALY*: RALY heterogeneous nuclear ribonucleoprotein
RJF: Red junglefow
*RYR2*: Ryanodine receptor 2
*SLC23A2*: Solute carrier family 23 member 2
*SLC2A14*: Solute carrier family 2 member 14
SNP: Single nucleotide polymorphism
SV: Structural variation
VEP: Variant effect predictor
YFC: Yellow-feathered chicken
