# Supplementary material for: Genome-wide genetic structure and selection signatures for color in 10 traditional Chinese yellow-feathered chicken breeds
Source: BMC Genomics. 2020 Apr 20;21:316. doi: 10.1186/s12864-020-6736-4 (PMC7171827; doi:10.1186/s12864-020-6736-4)
Supplement: Supplementary file 4 — Additional file 4: Fig. S1 Constitution of clean sequencing reads of all 110 chicken genomes produced in this study. Fig. S2 Summary of the average sequencing coverage of all 110 chicken genomes generated in this study. Fig. S3 Circos plot depicting the genomic variants landscape in each chromosome. Fig. S4 Annotation of the clean genomic SNPs of all 110 chickens sequenced in this study. Fig. S5 Transition-transversion analysis of the clean SNPs of all 110 chicken genomes sequenced in this study. Fig. S6 Annotation of the clean InDels of all 110 chicken genomes sequenced in this study. Fig. S7 Summary of the structural variations (SVs) and copy number variations (CNVs) in all 110 chicken genomes generated in this study. [file 12864_2020_6736_MOESM4_ESM.docx]

**Additional file 4**


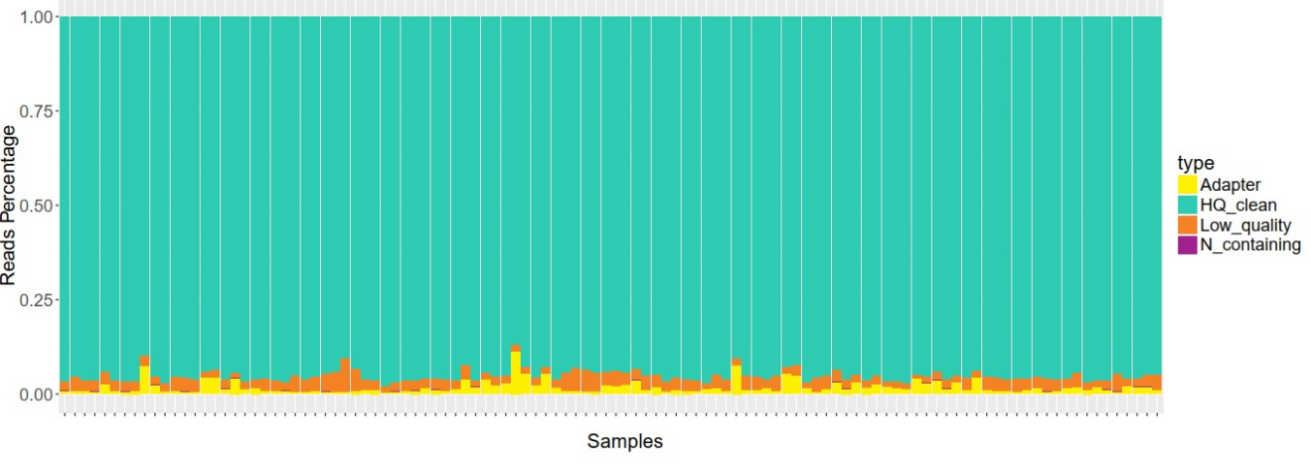


**Fig. S1 Constitution of clean sequencing reads of all 110 chicken genomes produced in this study.** Adapters (yellow) indicate reads with sequencing adapters below the filtering threshold, HQ_clean (green) represents high quality reads, Low_quality (orange) indicates reads with a low number of low quality bases, N_containing (purple) indicates reads with a low degree of N bases.


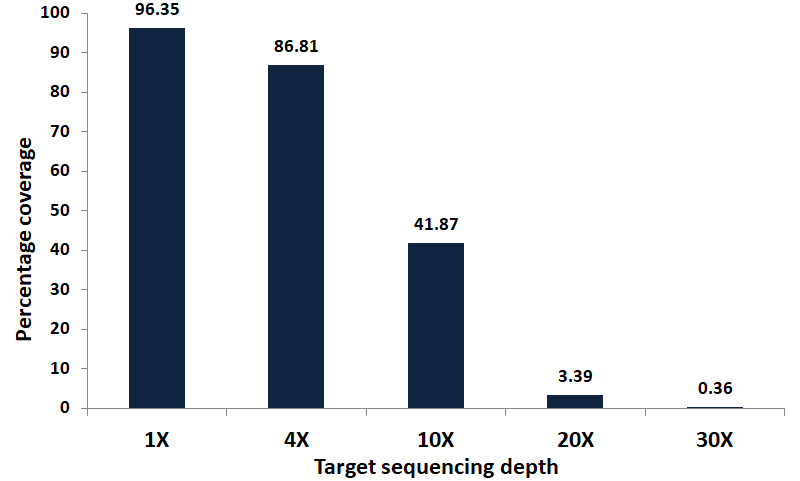


**Fig. S2 Summary of the average sequencing coverage of all 110 chicken genomes generated in this study.** Coverage statistics are computed up to the 30X target depth with reference to the Galgal5 reference genome.


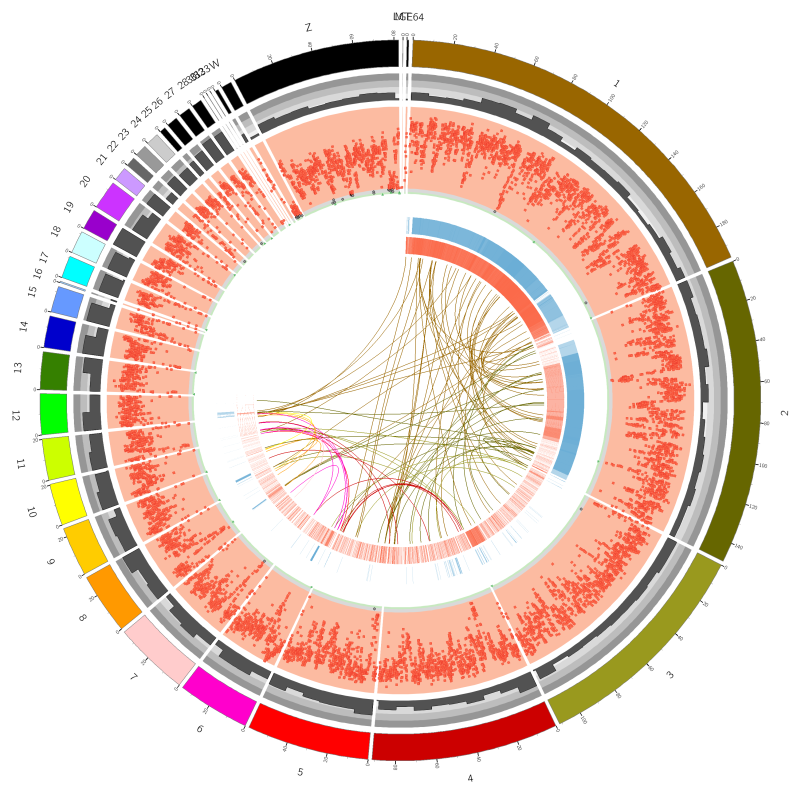


**Fig. S3 Circos plot depicting the genomic variants landscape in each chromosome.** Different loops from outside to inside summarize the length of each chromosome (unit: Mb), the gene density, the SNP density (SNP density > 0.0015 is marked by a red square, 0.0005 < SNP density ≤ 0.0015 by gray circle, and SNP density ≤ 0.0005 by a green triangle), the positions of INS (structural variation of the insertion type), the positions of INV (structural variation of the inverted type) on the chromosome, the positions of ITX (structural variation of the intrachromosomal translocation type). The lines of the inner circle indicate positions of CTX (structural variation of the interchromosomal translocation type) on the two chromosomes.

**Fig. S4 Annotation of the clean genomic SNPs of all 110 chickens sequenced in this study.** Proportions of SNPs are classified according to: (**a**) the genomic locations in which they occur; and (**b**) the genetic coding attributes.


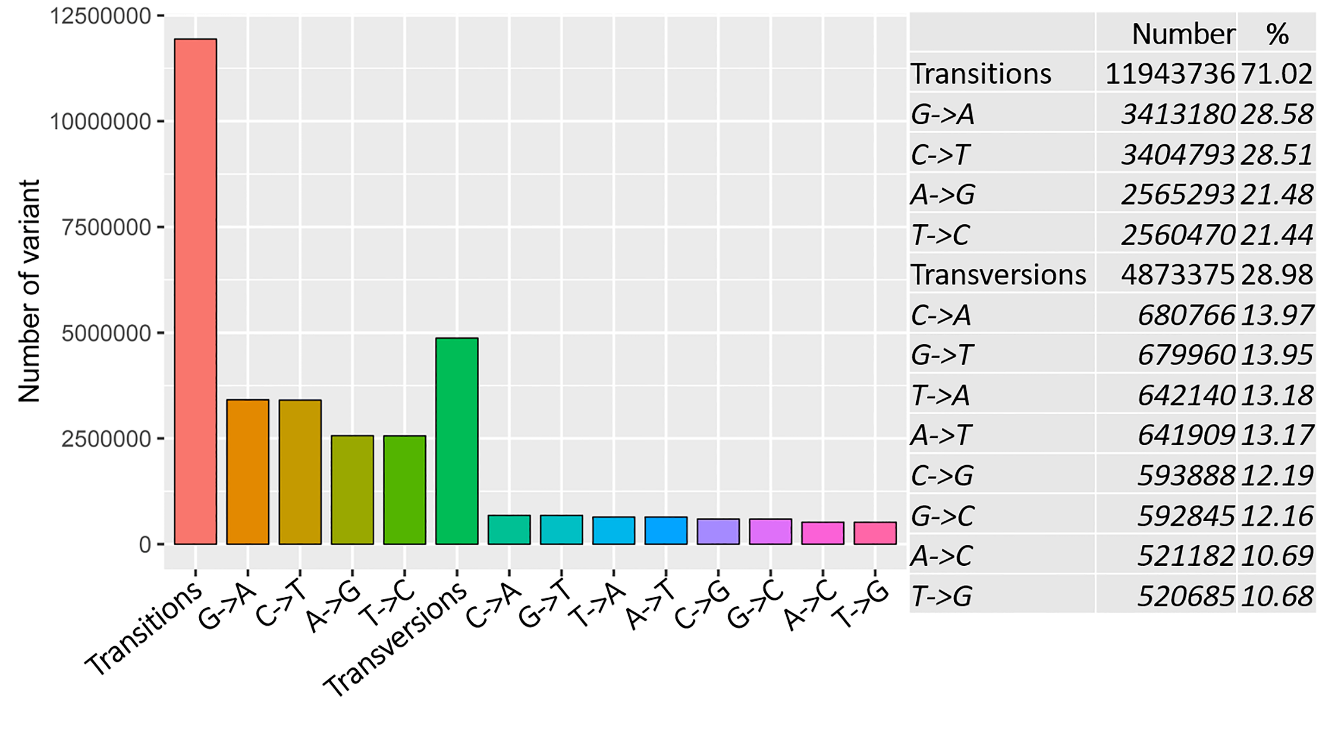


**Fig. S5 Transition-transversion analysis of the clean SNPs of all 110 chicken genomes sequenced in this study.** Bars represent the total number of transitional SNPs (red) followed by the individual base transitions types, and the total number of transversional SNPs (green) followed by the individual base transversion types. Numbers and proportions of the two groups of variants are their constituent variants are shown.

**Fig. S6 Annotation of the clean InDels of all 110 chicken genomes sequenced in this study.** Proportions of InDels are classified according to: **a**. the genomic locations in which they occur; and **b**. the genetic coding attributes.


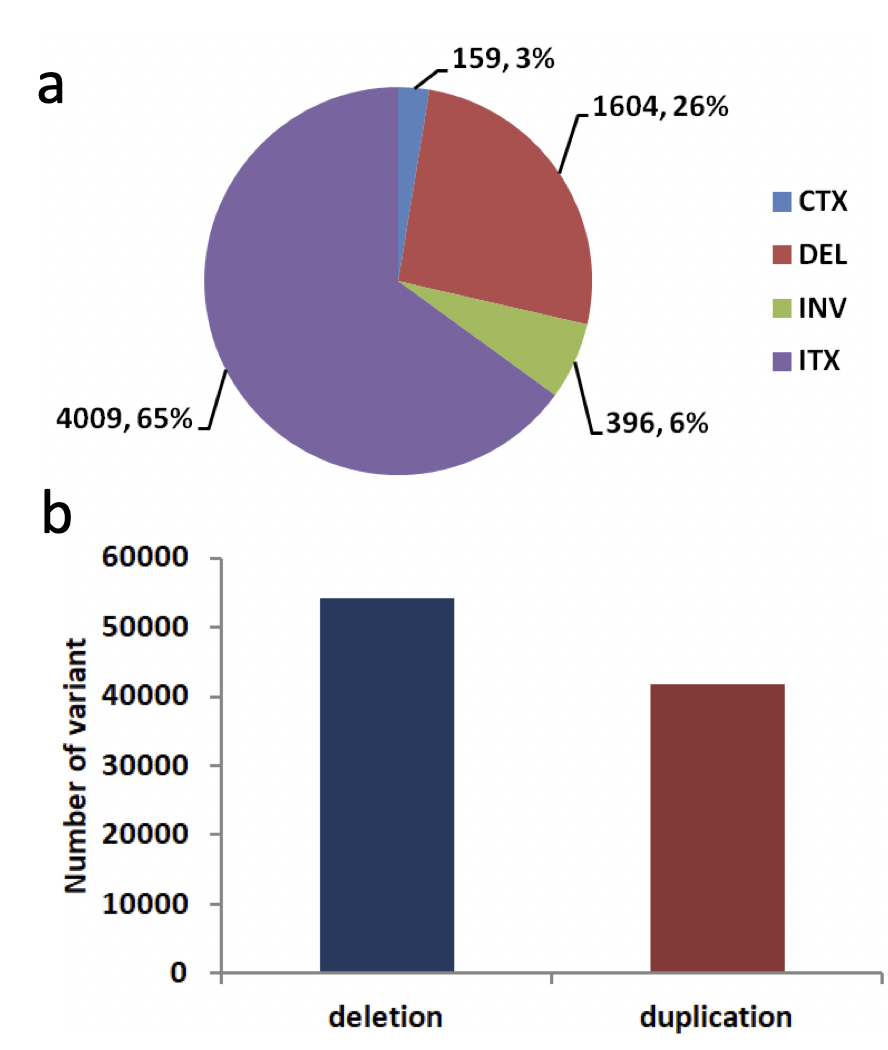


**Fig. S7 Summary of the structural variations (SVs) and copy number variations (CNVs) in all 110 chicken genomes generated in this study.** (**a**) Proportion of different SV types. CTX (interchromosomal translocation), DEL (deletion), INV (inversion), and ITX (intrachromosomal translocation). (**b**) Number of CNVs of both deletion and duplication types identified in the dataset.
